# MDM2 Inhibitor, Nutlin 3a, Induces p53 Dependent Autophagy in Acute Leukemia by AMP Kinase Activation

**DOI:** 10.1371/journal.pone.0139254

**Published:** 2015-10-06

**Authors:** Gautam Borthakur, Seshagiri Duvvuri, Vivian Ruvolo, Durga Nand Tripathi, Sujan Piya, Jared Burks, Rodrigo Jacamo, Kensuke Kojima, Peter Ruvolo, Juan Fueyo-Margareto, Marina Konopleva, Michael Andreeff

**Affiliations:** 1 Section of Molecular Hematology and Therapy; Departments of Leukemia and Stem Cell Transplantation, UT MD Anderson Cancer Center, Houston, Texas, United States of America; 2 Centre for Translational Cancer Research, Institute for Biosciences & Technology, Texas A&M Health Science Center, Houston, Texas, United States of America; 3 Department of Neuro-oncology, UT MD Anderson Cancer Center, Houston, Texas, United States of America; Rush University Medical Center, UNITED STATES

## Abstract

MDM2 (mouse double minute 2) inhibitors that activate p53 and induce apoptosis in a non-genotoxic manner are in clinical development for treatment of leukemias. P53 can modulate other programmed cell death pathways including autophagy both transcriptionally and non-transcriptionally. We investigated autophagy induction in acute leukemia by Nutlin 3a, a first-in-class MDM2 inhibitor. Nutlin 3a induced autophagy in a p53 dependent manner and transcriptional activation of AMP kinase (AMPK) is critical, as this effect is abrogated in AMPK -/- mouse embryonic fibroblasts. Nutlin 3a induced autophagy appears to be pro-apoptotic as pharmacological (bafilomycin) or genetic inhibition (BECLIN1 knockdown) of autophagy impairs apoptosis induced by Nutlin 3a.

## Introduction

MDM2 (HDM2 in humans), a E3 ubiquitin ligase, is a key regulator of p53 function through its role in proteasomal degradation of p53.[1, 2] Small molecule inhibitors of MDM2 that can restore wild type p53 activity in a non-genotoxic manner and thereby activate apoptotic cell death, are in clinical development as treatment of cancers including leukemias and have shown early clinical activity.[3, 4] Nutlin 3a and its analogs are first-in-class MDM2 inhibitors [3]. Preclinical studies with Nutlin 3a, as well as studies conducted by our group with patient samples from the first clinical trial with an analog of Nutlin 3a, have shown that consistent with their mechanism of action, Nutlin 3a and its analogs activate apoptosis program in acute myeloid leukemia (AML) cells only in the context of intact p53.[4, 5]

While the roles of p53 in apoptosis and cell cycle have been the ones most studied in the context of cancer, p53 can also play significant roles in programmed cell death (PCD) pathways other than apoptosis and in cellular adaptation to metabolic and environmental stress.[6] Autophagy is considered type II PCD and is such an adaptive mechanism with a Janus-like role in cell survival and cell death.[7, 8] While adaptive autophagy may sustain survival of cell including cancer cell, sustained autophagy can result in cell death. P53 in turn has a Janus-like effect on autophagy as it can transcriptionally or non-transcriptionally activate or inhibit autophagy. The effect of p53 on autophagy is dependent on the nature of the stress stimulus and subcellular location of p53.[9] While nuclear p53 can activate an autophagy program,[10] cytoplasmic p53 may inhibit autophagy.[11, 12] To add to the complexity, Atg7, a core autophagy protein, can in turn influence the transcriptional program of p53 in response to metabolic stress. In the absence of Atg7, p53 response to nutrient deprivation changes from one of cell-cycle arrest to a predominantly pro-apoptotic one.[13] Finally, genotoxic activation of p53 upregulates autophagy intiating kinase ULK1 and autophagy in this context enhances cell death.[14]

We set out to study the effect of non-genotoxic activation of p53 through MDM2 inhibition on autophagy in acute leukemia. In addition we wanted to identify key molecules involved in the process and the biological impact of autophagy in this context.

## Methods

### Reagents

Nutlin 3a was kindly provided by Discovery Oncology, Roche Research Center, Hoffmann-La Roche Inc., Nutley, NJ. A stock solution of 5mM in dimethyl sulfoxide (DMSO) was stored at -20°C. The final DMSO concentration in the medium did not exceed 0.1% (vol/vol). Autophagy inhibitor, Bafilomycin, was obtained from Sigma (St. Louis, MO). Antibodies to AMPKα (#2532), AMPK β (#4182), Beclin 1 (# 3738), p53 (#2527), Atg12 (#2010), LC3-B (#2775), LKB1 (#3050) were obtained from Cell Signaling (Danvers, MA) and antibody to p62 (sc–28359) was obtained from Santa Cruz (Dallas, TX).

### Cell lines and lentivirus

OCI-AML3 cells [15] (human leukemia cell line kindly provided by Dr. Mark Minden, Ontario Cancer Center, Canada) and OCI-AML–3 cells stably expressing shRNA targeting p53 and vector control [16] (kind gifts from Dr. Paul Corn, University of Texas MD Anderson Cancer Center), mouse embryonic fibroblasts (MEF) ^wt/wt^, ^AMPK -/-^ [17] (kind gifts from Dr. Juan Fueyo-Margareto, Neuro-oncology, MD Anderson Cancer Center) were cultured in RPMI 1640 medium containing 10% heat-inactivated fetal calf serum (FCS). HL60, HEK-293T and REH cells were obtained from the ATCC (Manassas, VA). Phoenix Amphotrophic retrovirus packaging cells were obtained from Orbigen (San Diego, CA). A set of 7 shRNAmirs each targeting BECLIN 1 plus non-silencing control lentiviral vector were obtained from Open Biosystems (Huntsville, AL). Lentiviral packaging plasmids MD2.G (plasmid 12259) and psPAX2 (plasmid 12260), both constructed by the laboratory of Didier Trono, plus retroviral vectors pMKO.1 puro p53 shRNA#2 (plasmid 10672), pMKO.1 puro GFPshRNA (plasmid 10675), and pBABE-puro mCherry-EGFP-LC3B (plasmid 22418) (constructed in the laboratory of Jayanta Debnath) were obtained from Addgene (Cambridge, MA).

### Lentiviral transduction

Each lentiviral vector was transiently cotransfected with an equimolar mix of the packaging plasmids into HEK-293T cells using Lipofectamine 2000 (Invitrogen, Carlsbad, CA) as directed by the manufacturer. Lentiviral supernatants were harvested 48 h post transfection. OCI-AML3 or REH cells were re-suspended in virus stock at a concentration of 0.8 x 106 per ml and spinoculated 90 minutes at 30°C at 1800×g. Infected cells were then washed with growth medium, and allowed to double once and then selected with puromycin (InvivoGen, San Diego, CA). Puromycin-resistant pools of cells were assessed for BECLIN 1 knockdown by Western analysis. Retroviral transduction was performed by a similar method, although in this case retrovirus was generated by transfection of Phoenix Ampho packaging cells by each retroviral vector.

### Western Blots

Signals were detected using Odyssey Infrared Imaging System (LI-COR Biosciences, Lincoln, Nebraska), and quantitated using Odyssey Software version 3.0 (LI-COR Biosciences). β-Actin was used as a loading control.

### Autophagy detection by confocal microscopy

OCI-AML3 cells transduced with pBABE-puro mCherry-EGFP-LC3B construct were treated with DMSO or Nutlin 3a and examined using a FV1000 laser confocal microscope to identify autophagic puncta.

### Autophagy/apoptosis quantitation by flow cytometry

Mono-Dansyl Cadaverine (MDC) has been used to label autophagic vacuoles in cells for visualization by microscopy.[18] We used MDC labelling and Annexin V binding to measure the number of cells undergoing autophagy and apoptosis respectively. Briefly 0.25x10^6^ cells were either treated with DMSO or Nutlin 3a for up to 120 hrs. The cells were then harvested, washed twice with PBS. They were re-suspended in 100 μL Annexin binding buffer containing 50 μM MDC (Sigma) and incubated in the dark for 45 minutes at 37°C. Then FITC conjugated Annexin V antibody (Roche Diagnostic, Indianapolis, IN) was added and further incubated for 15 minutes. The cells were then washed twice in Annexin binding buffer and re-suspended in 200 μL Annexin binding buffer and analyzed by flow cytometry. The cells were analyzed in a BectonDickenson LSR-II analytical flow cytometer, with the FITC on FL–1 on the log scale and the MDC on FL–8 on the linear scale. Countbright beads (Molecular Probes) were used to quantitate the number of negative, single positive and double positive cells.

### Transmission Electron Microscopy

Samples were fixed with a solution containing 3% glutaraldehyde plus 2% paraformaldehyde in 0.1 M cacodylate buffer, pH 7.3, for 1 hour. After fixation, the samples were washed in 0.1 M cacodylate buffer and treated with 0.1% Millipore-filtered buffered tannic acid, post-fixed with 1% buffered osmium tetroxide for 30 min, and stained en bloc with 1% Millipore-filtered uranyl acetate. The samples were washed several times in water, then dehydrated in increasing concentrations of ethanol, infiltrated, and embedded in LX–112 medium. The samples were polymerized in a 60 C oven for 2 days. Ultrathin sections were cut in a Leica Ultracut microtome (Leica, Deerfield, IL), stained with uranyl acetate and lead citrate in a Leica EM Stainer, and examined in a JEM 1010 transmission electron microscope (JEOL, USA, Inc., Peabody, MA) at an accelerating voltage of 80 kV. Digital images were obtained using AMT Imaging System (Advanced Microscopy Techniques Corp, Danvers, MA) at the Cancer Biology research laboratory facility.

### Statistical analyses

The Student *t*-test was used to analyze cell growth, and apoptosis data. A *P*-value ≤ 0.05 was considered statistically significant. All statistical tests were two-sided and the results are expressed as the mean two independent experiments of triplicate samples/experiments ± S.D./95% confidence intervals (error bars).

## Results

### Nutlin 3a induces autophagy in acute myelogenous leukemia cells

**Fig 1:**
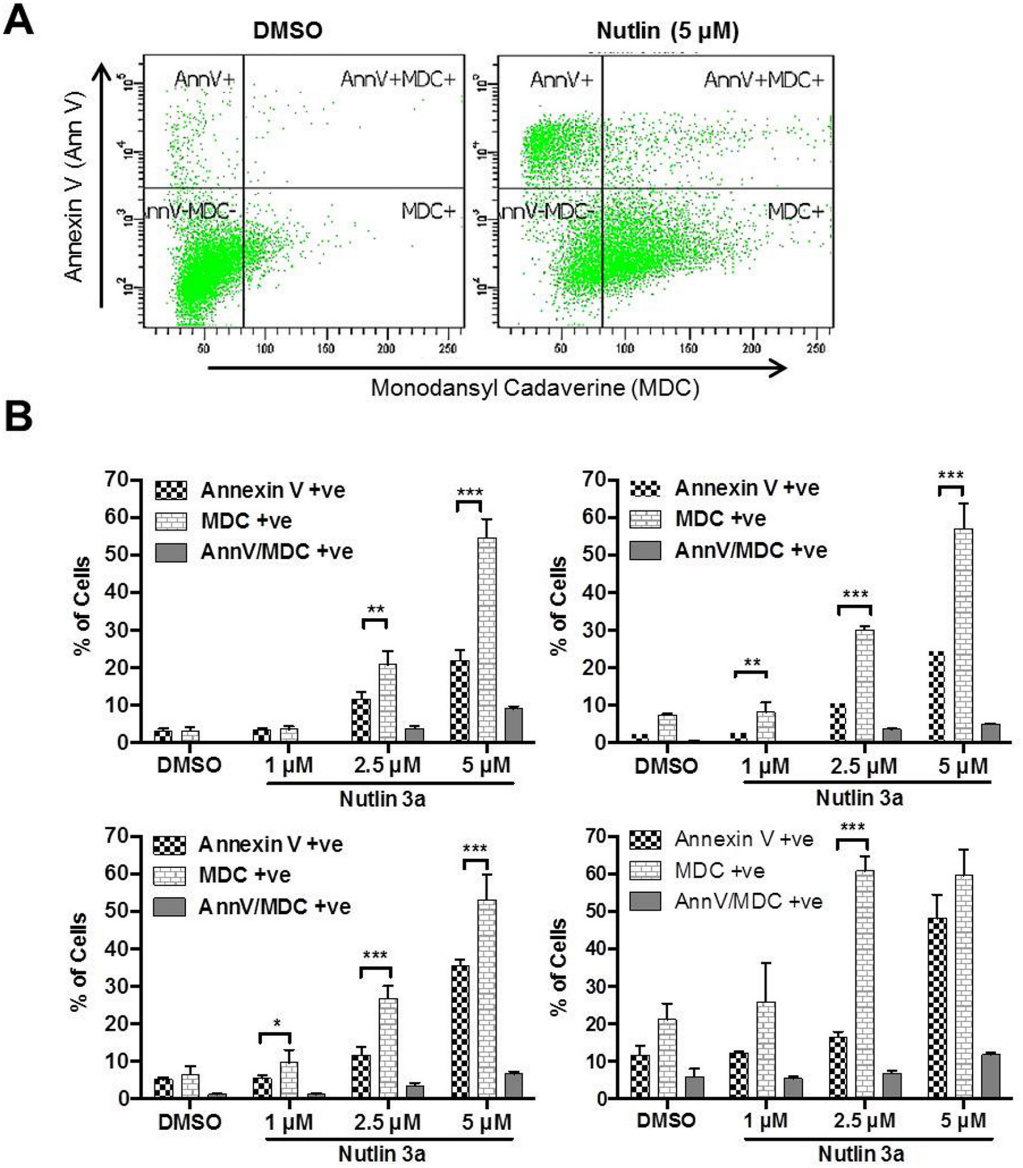
**A** OCI-AML3 cells were treated with Nutlin 3a (5 μM) or DMSO for 72 hrs, stained with MDC and AnnV and analyzed by flow cytometry for AnnV positive (apoptosis), MDC positive (autophagy) and dual positive cells. **B** Time and concentration dependent change in AnnV positive, MDC positive and dual positive OCI-AML3 cells treated with Nutlin 3a.

We used flowcytometry, confocal microscopy and Western blot analysis to demonstrate autophagy induction. OCI-AML3 cells were treated with DMSO or Nutlin 3a (1, 2.5 and 5 μmol) for 48, 72, 96 and 120 hrs. Cells were stained with Annexin V-APC and lysosomotropic dye MDC and analyzed by flow cytometry to detect apoptosis (Annexin V-APC binding), autophagy (MDC). Treatment with Nutlin 3a treatment increased the percentage of Annexin V positive, MDC positive and dual positive (MDS and Annexin v-APC) cells (Fig 1A) in a time (48–120 hrs) and dose dependent (1–5 μmol) manner. The percentage of cells with MDC staining was interestingly highest at a late time point of 120 hrs and at Nutlin 3a concentration of 2.5 and 5 μM (61 and 60% respectively versus 21% for DMSO treated control) (Fig 1B). Importantly, at each time point, the percentage of MDC positive cells was significantly higher than that of Annexin V positive cells; this was most evident at later time points of 96 and 120 hrs at a Nutlin 3a concentration of 2.5 μmol indicating sustained autphagy.

**Fig 2:**
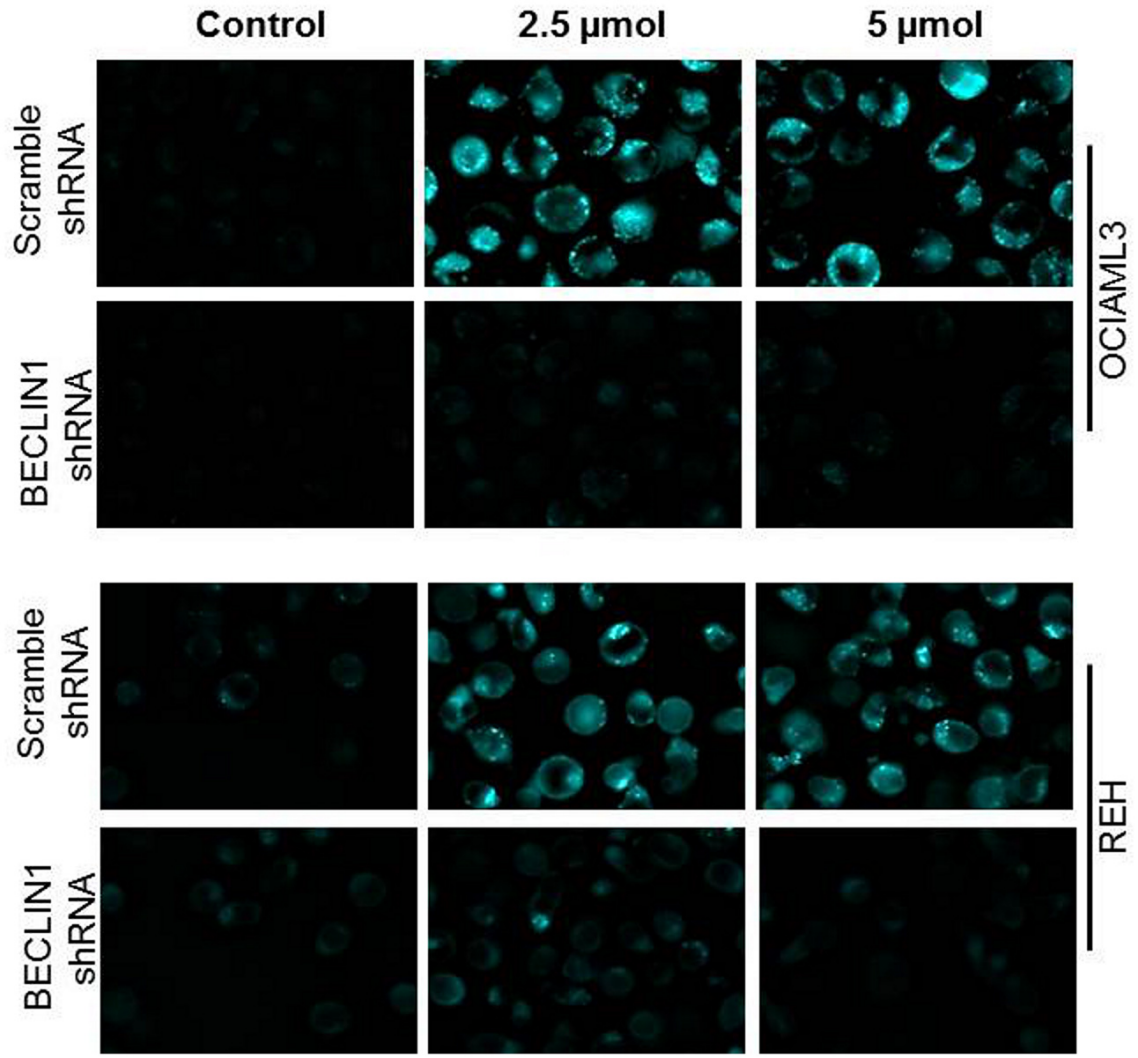
OCI-AML3 or REH (p53 wild type) cells stably expressing either control or Beclin1-silencing shRNA were treated with Nutlin 3a, stained with MDC and imaged with confocal microscopy for MDC positive ‘puncta’ representing autophagic vacuoles.

A key feature of the autophagy process is the formation of autophagic vacuoles. We used confocal fluorescence microscopy to visualize autophagic vacuole formation using MDC. OCI-AML3 cells treated with DMSO or Nutlin 3a (2.5 and 5 μM for 72 hrs.) were stained with MDC and imaged using fluorescence microscope. At both concentrations of Nutlin 3a, the number of MDC positive vacuoles increased compared to DMSO treated cells (Fig 2, Panel 1). To confirm that MDC positive puncta formation is indicative of autophagy, we treated OCI-AML3 cells stably expressing shRNA targeting BECLIN1, an essential autophagy gene, with Nutlin 3a for same-period time. MDC positive puncta formation was markedly diminished in BECLIN1 shRNA expressing OCI-AML3 cells (Fig 2, Panel 2), confirming that the formation of MDC positive puncta is indicative of autophagy. This was also confirmed using REH (acute lymphoblastic leukemia) cells transduced with control shRNA (Fig 2, Panel 3) or BECLIN1 shRNA (Fig 2, Panel 4).

**Fig 3:**
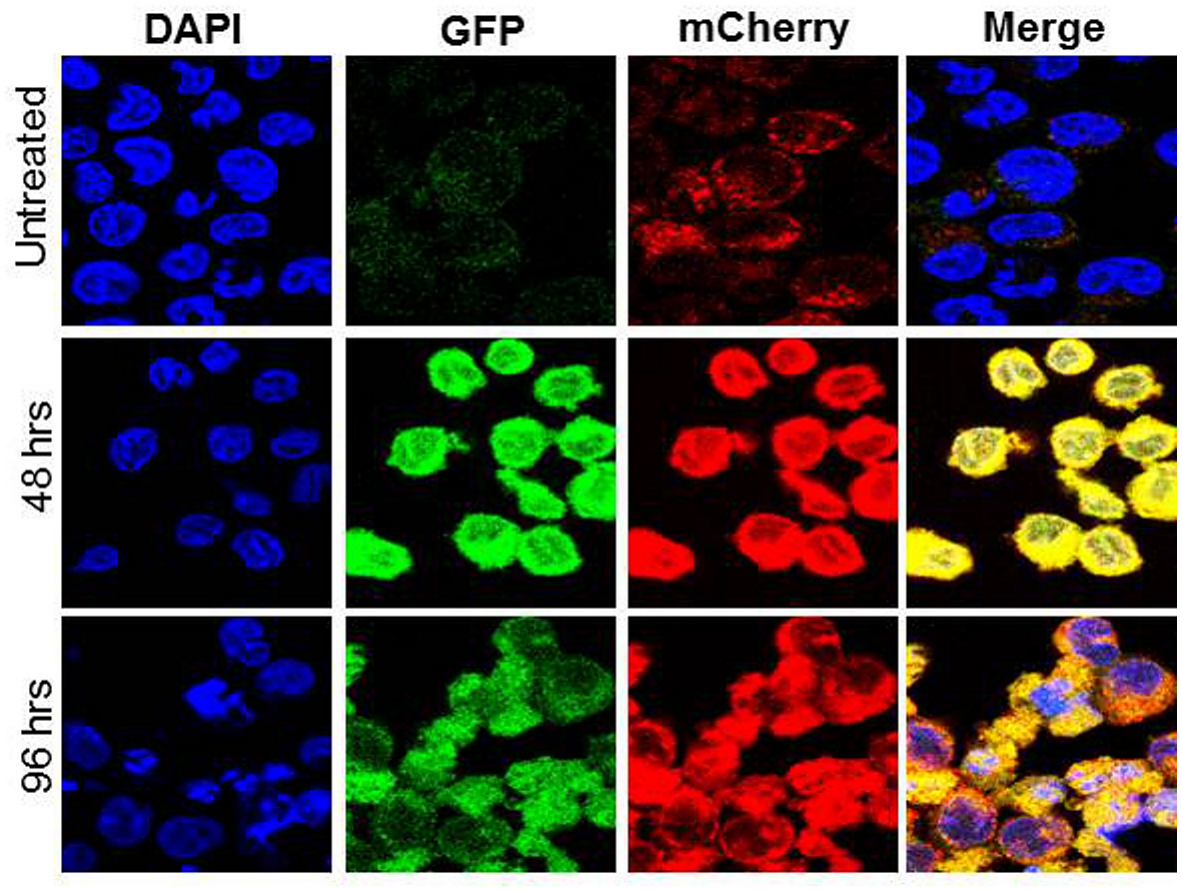
OCI-AML3 cells transduced with LC3-GFP-mCherry construct were treated with 5 μM Nutlin 3a and visualized by confocal microscopy. Increase in proportion of red ‘puncta’ at 96 hrs compared to 48 hrs (merge images) in Nutlin 3a treated cells indicate completion of autophagic flux.

Increased numbers of autophagic vacuoles in a cell could be the result of either increased formation of autophagic vacuoles or arrest of autophagic flux. The later actually is a state of impaired autophagy as here autophagic vacuoles fail to fuse with lysosomes to form autophagosomes. An mCherry-EGFP-LC3B reporter construct has been used for assessment of autophagic flux.[19] The GFP signal in this construct is unstable in the acidic environment resulting from fusion of autophagic vacuoles with lysosomes (autophagosome) while the mCherry signal persists. With induction of autophagy there is an initial increase in both green and red fluorescence (yellow in merged images), while completion of autophagic flux (formation of autophagosome) is accompanied by a proportional increase in red fluorescence (compared to yellow) in merged images. OCI-AML3 cells stably expressing the mCherry-EGFP-LC3B reporter construct were treated with Nutlin (2.5 μM). Confocal imaging showed time dependent increase in both green and red ‘puncta’ (yellow in merged images) suggesting ongoing and sustained autophagy after Nutlin 3a treatment. The proportion of red ‘puncta’ (in comparison to yellow indicating acidification of vacuoles) increased at 96 hrs compared to 48 hrs indicating time dependent completion of autophagic flux (Fig 3).

**Fig 4:**
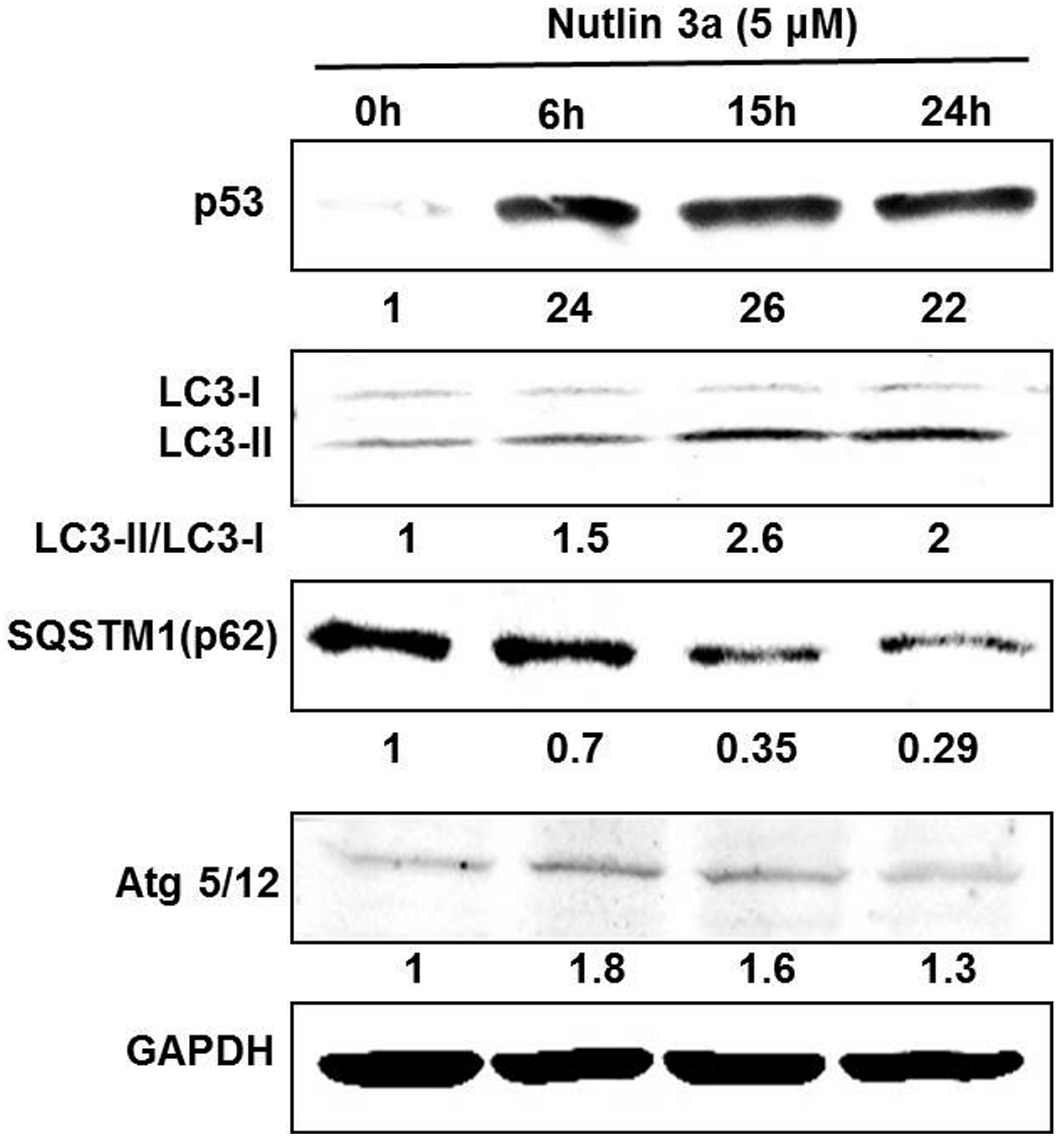
OCI-AML3 cells were treated with Nutlin 3a for indicated time and Western blots done for p53, LC3B, Atg 5/12, p62.

Finally induction of autophagy was assessed by Western blot analysis of OCI-AML3 cells treated with Nutlin 3a at 5 μM for 0,6,12 and 24 hrs. Western blots of whole cell lysates confirmed increased LC3-II formation, Atg 5/12 conjugation and decrease in p62 protein in a time dependent manner (Fig 4). As expected Nutlin 3a treatment also increased p53 protein level.

### Autophagy induced by Nutlin 3a is p53 dependent

**Fig 5:**
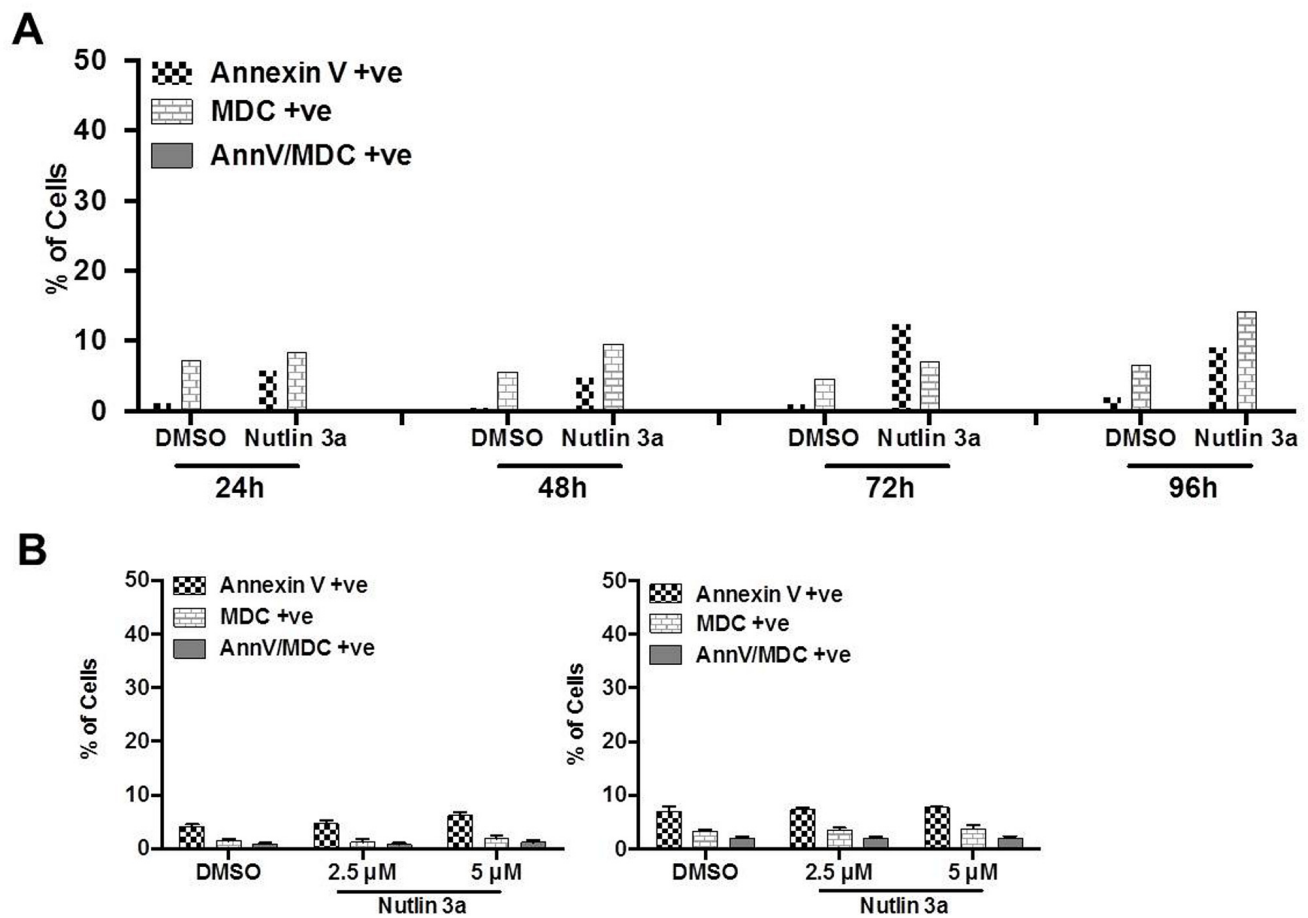
HL60 (A) or OCI-AML3 cells stably expressing shRNA silencing p53 (B) were treated with Nutlin 3a for 24–96 hrs and analyzed by flow cytometry for AnnV/MDC staining.

Work published from our laboratory has shown that Nutlin 3a induces apoptosis only in p53 WT AML cells, consistent with its mechanism of action. To test if autophagy induced by Nutlin 3a is also p53 dependent, HL60 (p53 null AML) cells and OCI-AML3 cells in which p53 was knocked down by lentiviral transduction of shRNA (OCI-AML3 p53 shRNA) (knockdown efficacy published previously)[20]) were treated with 5 μM Nutlin 3a for up to 96 hrs and stained with Annexin V and MDC. After 96 hrs of treatment with Nutlin 3a, only 9% HL 60 cells stained positive for MDC (versus 2% DMSO treated control) and 14% for Annexin V (versus 6.5% control) (Fig 5A), indicating that both Nutlin 3a induced apoptosis and autophagy are p53 dependent. Similarly only 3% of OCI-AML3 p53 shRNA cells were MDC positive (versus 4% with DMSO treatment) and 15% was Annexin V-APC positive (versus 11% with DMSO) (Fig 5B). Similar data were obtained using REH leukemia cells (acute lymphoblastic leukemia cell line with WT p53) and their p53 shRNA expressing counterparts (data not shown).

**Fig 6:**
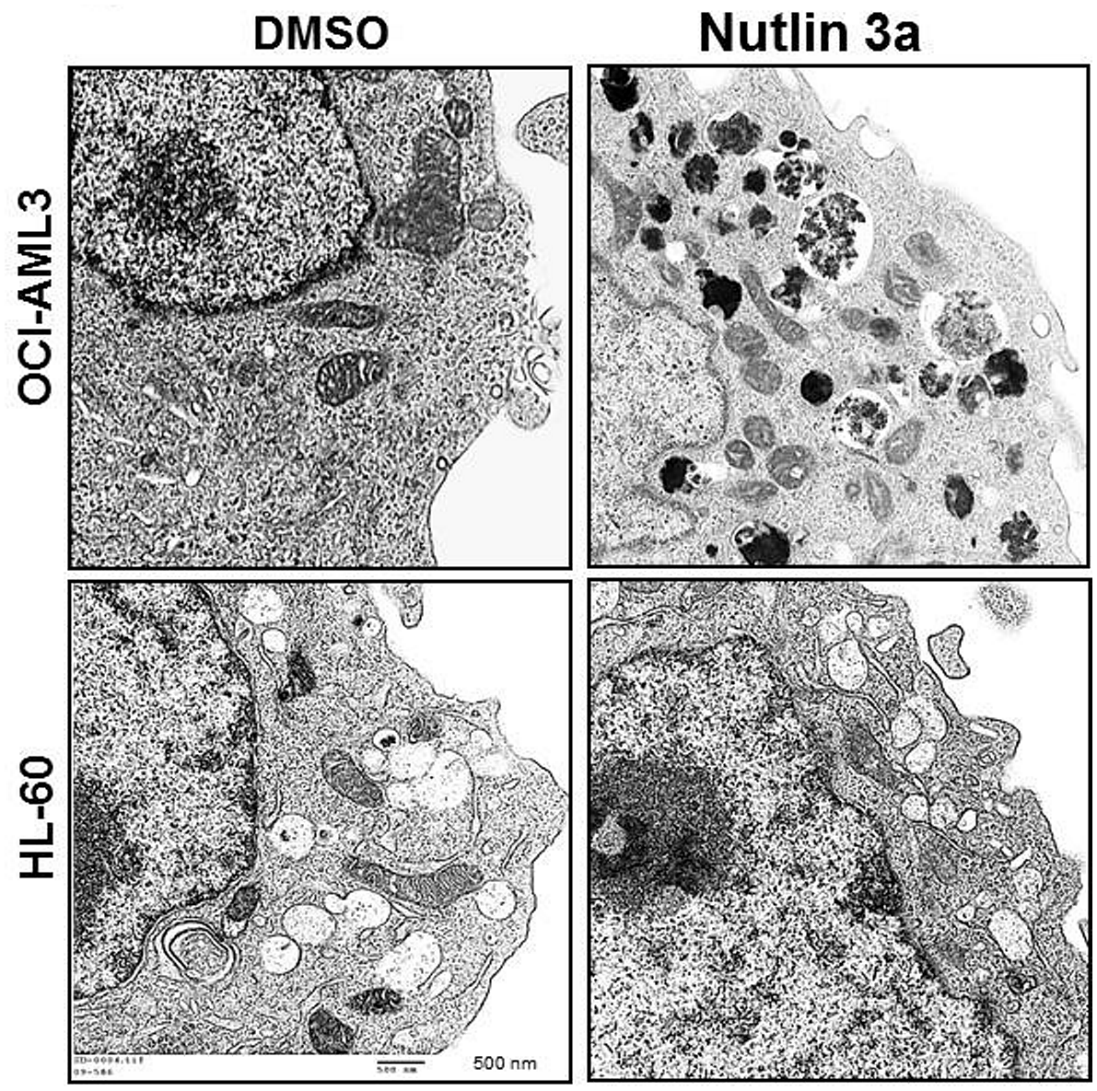
OCI-AML3 (p53 wild type) or HL–60 (p53 null) cells were treated with DMSO or Nutlin 3a for 72 hours and imaged using transmission electron microscopy. While OCI-AML3 cells showed numerous electron-dense ‘mitophagic’ vacuoles at 72 hrs of Nutlin 3a treatment, such vacuoles were absent in HL–60 cells (lower panel).

Transmission electron microscopy (TEM) is broadly used as a ‘gold standard’ test for autophagy and the presence of autophagic vacuoles containing electron dense mitochondria (mitophagy) is considered both a hallmark and evidence of autophagy by TEM. We treated OCI-AML3 cells with Nutlin 3a (5 μmol for 48 hrs) and performed TEM. Treated cells showed marked increase in ‘mitophagic’ vacuoles compared to DMSO treated controls (Fig 6 upper panel). To confirm that Nutlin 3a induced autophagy is p53 dependent, HL–60 (p53 null) cells treated with Nutlin 3a (5 μM for 48–96 hrs) were examined by TEM for autophagy. Compared to OCI-AML3 cells, HL60 cells showed remarkably low number of mitophagic vacuoles confirming p53 dependence of Nutlin 3a induced autophagy (Fig 6 lower panel).

### Inhibition of Nutlin 3a induced autophagy reduces apoptosis

**Fig 7:**
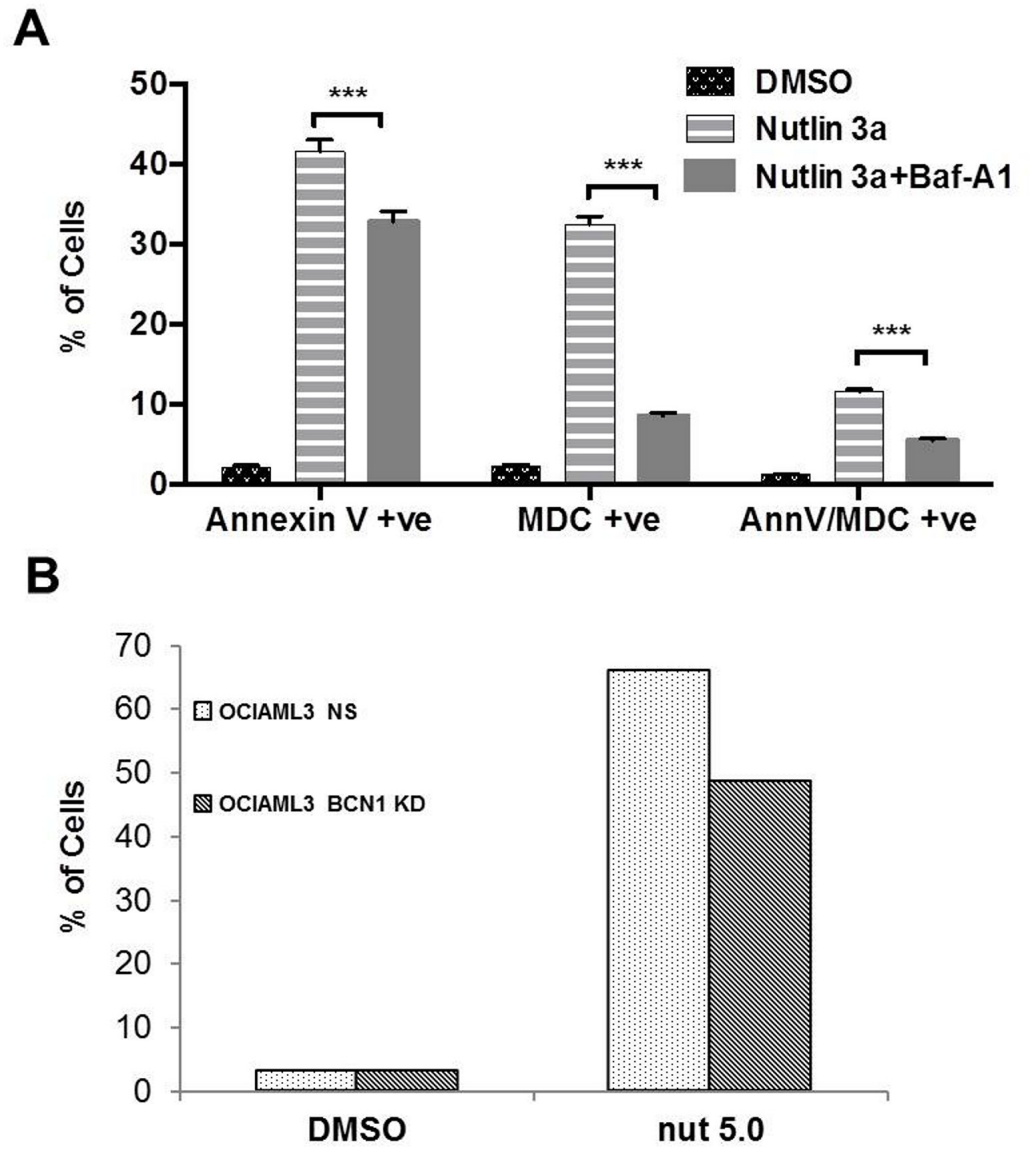
**A** OCI-AML3 cells were treated with Nutlin 3a (5 μmol) for 48 hrs alone or in combination with autophagy inhibitor bafilomycin added at 10 nM for last 2 hrs of the 48 hr period. Cells were stained with AnnV and MDC and analyzed by flow cytometry for AnnV, MDC or dual staining (AnnV+MDC). **B** OCI-AML3 cells stably expressing non-specific (NS) shRNA or shRNA targeting BCN1 (BCN1 KD) were treated with Nutlin 3a for 72 hours and apoptosis assessed by flow cytometry for AnnV. Results are average of two experiments.

To investigate the biological effect of blocking Nutlin 3a induced autophagy, OCI-AML3 WT cells were treated with Nutlin 3a (5 μM) for 48 hrs alone or in combination with autophagy inhibitor bafilomycin (10 nM) for last 2 hrs of incubation. Bafilomycin inhibits autophagy by inhibiting vacuolar ATPase to increase lysosomal pH. Bafilomycin treatment not only reduced Nutlin 3a induced autophagy as demonstrated by reduced MDC binding by flow cytometry but also reduced Nutlin 3a-induced apoptosis as evidenced by decreased Annexin V binding (Fig 7A). Similarly, apoptosis was lower after Nutlin 3a treatment (5 μmol for 72 hrs) in OCI-AML3 cells with stable knock-down of core autophagic protein Beclin 1 compared to control (68% vs 43% respectively) (Fig 7B). Copmpared to bafilomycin, loss of Beclin 1 is expected to block autophagy at an earlier time point.

### Nutlin 3a induces upregulation of AMPK

**Fig 8:**
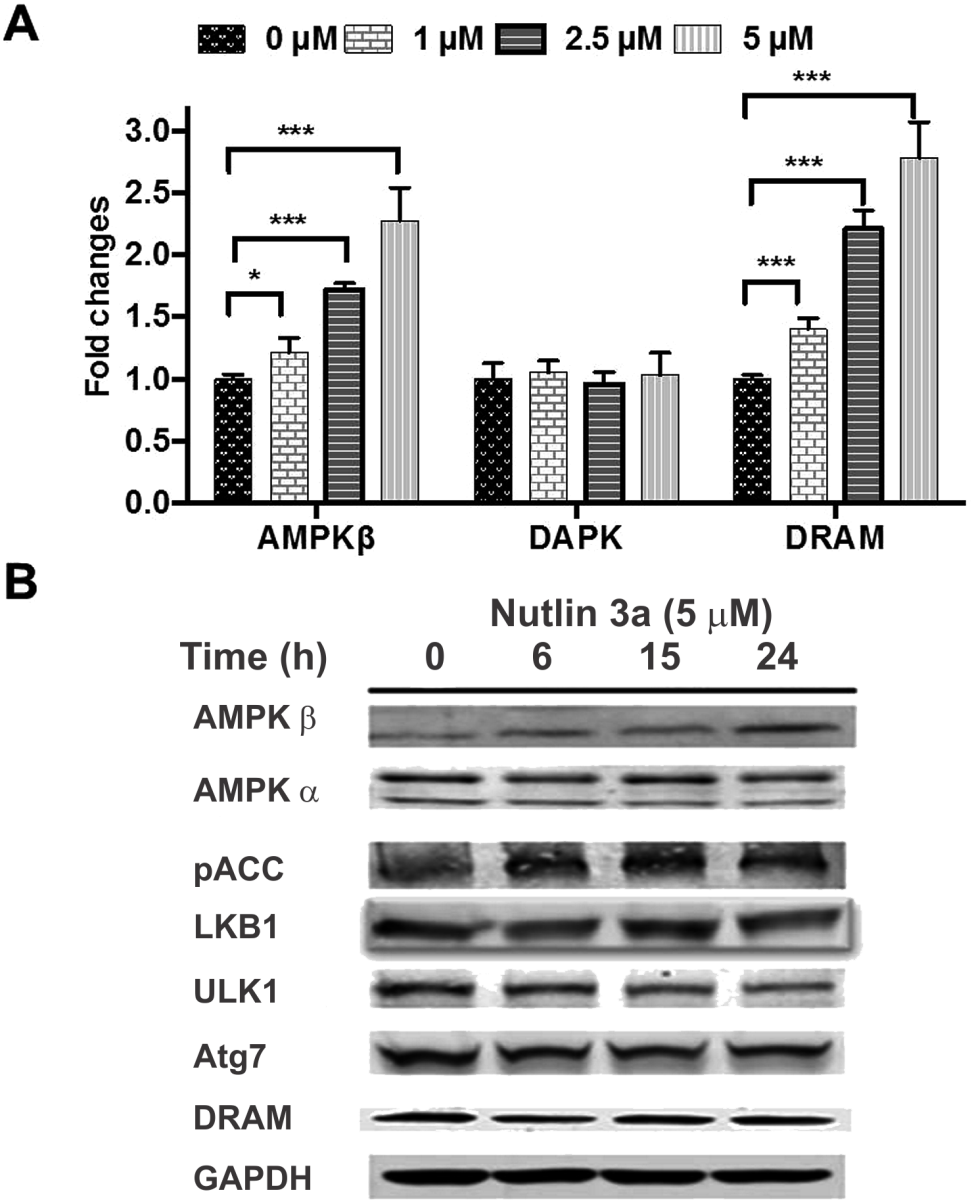
**A** OCI-AML3 cells were treated with Nutlin 3a (0, 1, 2.5 and 5 μM) for 12 hours, RNA was extracted and RT-PCR was performed for autophagy-related transcriptional targets of p53; DRAM, DAPK and AMP kinase beta (AMPK-β). **B** OCI-AML3 cells were treated with Nutlin 3a for 0–24 hours and Western blot was done for several autophagy related proteins

The beta subunit of AMPK (AMPK β), DRAM and DAPK are transcriptional targets of p53 that are commonly implicated in autophagy. Quantitative reverse transcriptase polymerase chain reaction (RT-QPCR) studies of OCI-AML3 cells after treatment with Nutlin 3a (0–5 μmol) for 12 hrs showed increase in transcripts of AMPK β and DRAM while that of DAPK remained unchanged (Fig 8A). Western blot analysis confirmed increase of AMPK β after treatment with Nutlin 3a but neither that of DRAM (Fig 8B) nor of the α subunit of AMPK showed any change. The increase in AMPK β protein is expected to stabilize the AMPK complex and enhance its activity. Acetyl coenzyme carboxylase (ACC) is the immediate downstream target of AMPK, and phosphorylated ACC was increased after OCI-AML3 cells were treated with Nutlin 3a (Fig 8B), confirming increased AMPK activity. The level of LKB1, an upstream kinase of AMPK, was not increased with Nutlin 3a treatment. ULK1 phosphorylation at serine 317 by AMPK activates autophagy while its phosphorylation at serine 737 by mTROC1 inhibits autophagy. Serine 317 phosphorylation increased in OCI-AML3 cells treated with Nutlin 3a while serine 757 phosphorylation was unchanged (Fig 8B), again confirming activation of AMPK by Nutlin 3a.

### AMPK is necessary for Nutlin 3a induced autophagy

**Fig 9:**
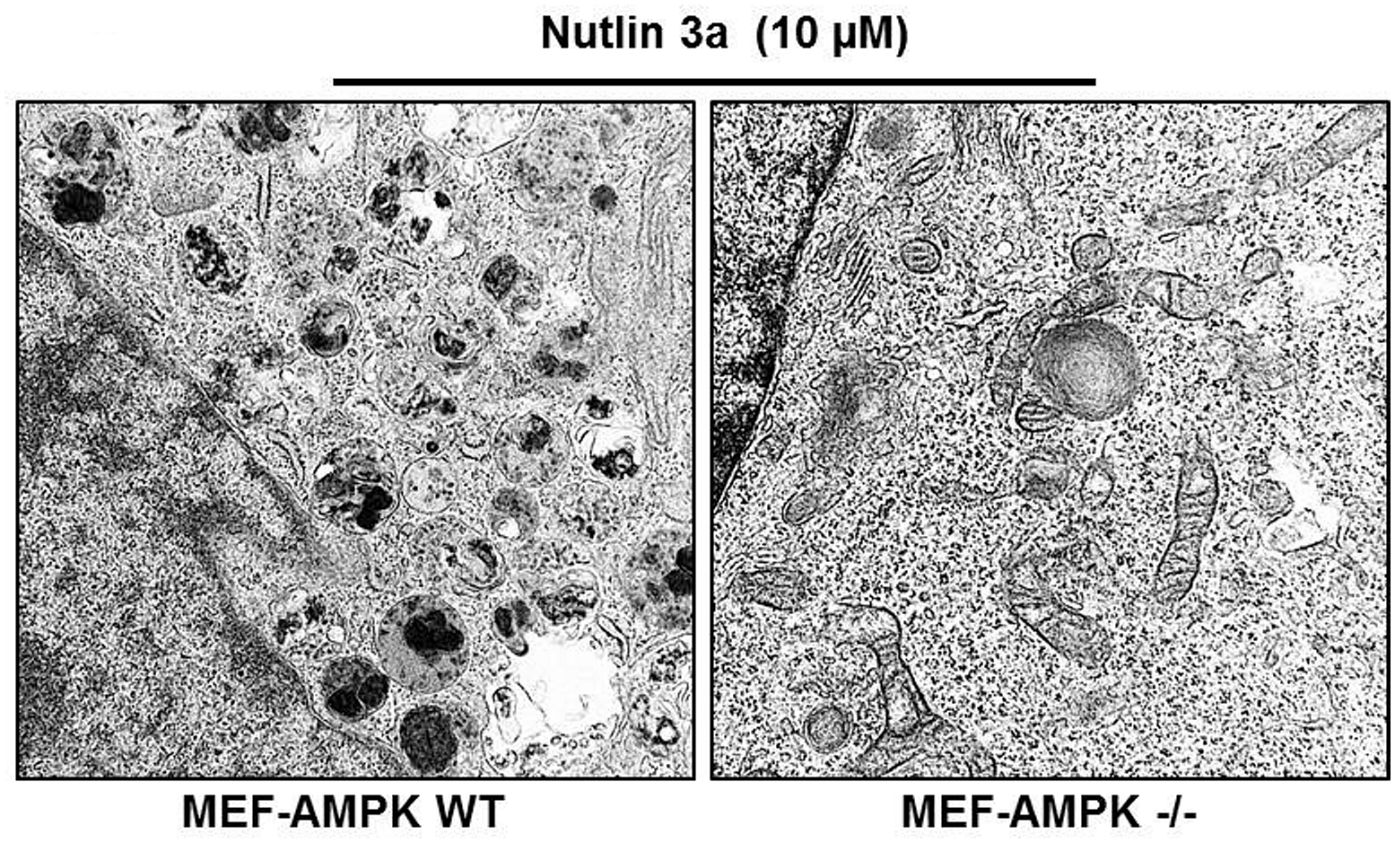
Mouse embryonic fibroblasts (wild type or AMPK ^-/-^) were treated with 10 μM Nutlin 3a for 72 hours and examined by transmission electron microscopy for ‘mitophagic’ vacuoles.

To examine the role of AMPK in Nutlin 3a-induced autophagy, mouse embryonic fibroblasts (MEF) AMPK ^wt/wt^ or AMPK ^-/-^ were treated with Nutlin 3a (10 μmol) for 48–96 hrs and examined by TEM for ‘mitophagy’. The number of mitophagic vacuoles was substantially higher in AMPK ^wt/wt^ MEFs compared to AMPK ^-/-^ MEFs (Fig 9). This finding confirms the essential role for AMPK in Nutlin 3a-induced autophagy.

## Discussion

This report demonstrates that Nutlin 3a induces sustained autophagy in AML cells in a p53-dependent manner and that transcriptional upregulation of AMPK is a key mediator of that process. Pharmacologic or genetic inhibition of autophagy in this context appears to interfere with apoptosis.

In autophagy studies, it is important to demonstrate completion of autophagic flux including fusion of lysosome to autophagic vacuole leading to its acidification; the final step in the process. Blockades of the later phases of autophagy prior to lysosomal fusion may also show apparent increase in autophagic ‘puncta’ or vacuoles while in reality autophagy is impaired.[21] Our experiments using an LC3-GFP-m Cherry construct confirms increased autophagic flux in AML cells with Nutlin 3a treatment. This is also confirmed by TEM studies (mitophagy) and immuno blot results of LC3 lipidation, Atg 5/12 conjugation and p62 degradation.

The β subunit of AMPK facilitates the hetero-trimeric association of the AMPK subunits enhancing the catalytic function of the complex that is comprised of α, β, γ sub-units [22–26] and is a transcriptional target of p53.[27] AMPK can activate autophagy by several means. Phosphorylation of ULK1 (Atg1 in yeast) at serine 317 by AMPK releases ULK1 from the inhibitory influence of mTORC1 and initiates autophagy.[28] Phosphorylation of TSC2 and Raptor by AMPK also suppresses mTORC1 activity resulting in the same effect on autophagy.[27] Though additional autophagy related proteins are known to be transcriptional targets of p53, our data with AMPK ^-/-^ MEFs suggest that in the context of Nutlin 3a, AMPK plays a major role in autophagy induction. DRAM on the other hand, is a lysosomal protein which is also a transcriptional target of p53 and modulates autophagy.[29] In our experiments, DRAM transcript levels were high in Nutlin 3a treated AML cells, but we could not confirm an increase in protein levels. We cannot rule out a potential role of DRAM in Nutlin 3a induced autophagy.

Recent reports implicate upregulation of ULK1 in p53 mediated autophagy after genotoxic damage with camptothecin and etoposide.[14] Our data on the other hand suggest that AMPK, a kinase upstream of ULK1 in canonical autophagy induction, is a critical mediator of autophagy after non-genotoxic activation of p53 through MDM2 inhibition. While we could not demonstrate any increase of ULK1 protein levels after Nutlin 3 a treatment, increased pro-autophagic S317 phosphorylation of ULK1, a phosphorylation event mediated by AMPK corroborates the role of AMPK in Nutlin 3a induced autophagy. Our results indirectly suggest that autophagy inducers could increase Nutlin 3a’s apoptotic effect. Therapeutic agents that directly modulate autophagy are limited. Inhibitors that target PI3K or Akt can induce autophagy indirectly by their effects on mTORC1, TSC2, etc. and can be potential therapeutic partners to MDM2 inhibitors. MTOR/Akt inhibitors are synergistic with MDM2 inhibitors [16] in inducing apoptosis in leukemias but whether this synergy is autophagy related has not been determined. LKB1, the upstream kinase for AMPK, is a target of metformin and its analogs, and this class of drugs may also synergize with MDM2 inhibitors in inducing pro-apoptotic autophagy.

In the field of therapeutic targeting of autophagy in cancer, there is increased awareness that the pro versus anti-apoptotic role of autophagy is context dependent and may vary with therapeutic agents.[30–33] This is highlighted by the fact that chloroquine and its analogs potentiate chemotherapy effects by inhibiting autophagy.

In summary, MDM2 inhibitor Nutlin 3a potently induces autophagy. While clinical development of several MDM2 inhibitors is in progress in cancer therapy, the biological impact of autophagy in their responses need to be explored.

## Supporting Information

S1 File OCI AML3 WT or p53 KD autophagy apoptosis with Nutlin. (XLS)

S2 File Nutlin with or without bafilomycin apoptosis autophagy. (XLS)

S3 File DRAM DAPK AMPK beta QPCR. (XLS)
